# 3,5-Dimethyl-4-nitroso-1*H*-pyrazole

**DOI:** 10.1107/S1600536811033794

**Published:** 2011-08-31

**Authors:** Inna Safyanova, Nikolay M. Dudarenko, Vadim A. Pavlenko, Turganbay S. Iskenderov, Matti Haukka

**Affiliations:** a Department of Chemistry, Kiev National Taras Shevchenko University, Volodymyrska Str. 64, 01601 Kiev, Ukraine; b Department of Chemistry, University of Joensuu, PO Box, 111, FI-80101 Joensuu, Finland

## Abstract

In the unit cell of the title compound, C_5_H_7_N_3_O, there are two conformers (*A* and *B*) which differ in the position of the oxime group with respect to the protonated pyrazole nitro­gen (*trans* in the *A* conformer and *cis* in the *B* conformer) and in the geometric parameters. The oxime group exists in the nitroso form in both conformers. In the crystal, mol­ecules are linked by inter­molecular N—H⋯O and N—H⋯N hydrogen bonds into zigzag-like chains along the *b* axis.

## Related literature

For the use of pyrazole-based ligands, see: Mullins & Pecoraro (2008 ▶); Mukhopadhyay *et al.* (2004 ▶). For the magnetic properties of pyrazolate complexes, see: Aromi & Brechin (2006 ▶); Gatteschi *et al.* (2006 ▶). For the use of oxime substituents in the synthesis of polynuclear ligands, see: Petrusenko *et al.* (1997 ▶); Kanderal *et al.* (2005 ▶); Sachse *et al.* (2008 ▶); Moroz *et al.* (2010 ▶). For the use of 4-nitro­pyrazoles as ligands, see: Halcrow (2005 ▶). For related structures, see: Fletcher *et al.* (1997 ▶); Kovbasyuk *et al.* (2004 ▶); Mokhir *et al.* (2002 ▶); Sliva *et al.* (1997 ▶); Wörl, Fritsky *et al.* (2005 ▶); Wörl, Pritzkow *et al.* (2005 ▶). For the synthesis of the title compound, see: Cameron *et al.* (1996 ▶).
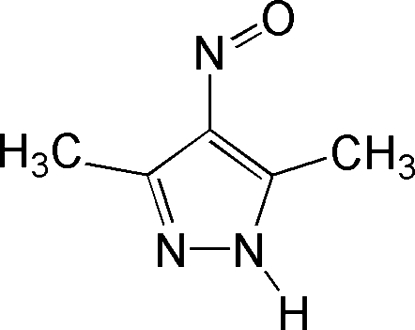


## Experimental

### 

#### Crystal data

C_5_H_7_N_3_O
*M* *_r_* = 125.14
Monoclinic, 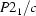

*a* = 4.0268 (2) Å
*b* = 15.3793 (7) Å
*c* = 19.6627 (9) Å
β = 94.613 (3)°
*V* = 1213.75 (10) Å^3^
*Z* = 8
Mo *K*α radiation
μ = 0.10 mm^−1^
*T* = 120 K
0.46 × 0.33 × 0.13 mm

#### Data collection

Nonius KappaCCD diffractometer
Absorption correction: multi-scan (*DENZO*/*SCALEPACK*; Otwinowski & Minor, 1997 ▶) *T*_min_ = 0.955, *T*_max_ = 0.987
9003 measured reflections
2747 independent reflections
1866 reflections with *I* > 2σ(*I*)
*R*_int_ = 0.040

#### Refinement

*R*[*F* ^2^ > 2σ(*F* ^2^)] = 0.041
*wR*(*F* ^2^) = 0.109
*S* = 1.03
2747 reflections
175 parameters
H atoms treated by a mixture of independent and constrained refinement
Δρ_max_ = 0.23 e Å^−3^
Δρ_min_ = −0.25 e Å^−3^

### 

Data collection: *COLLECT* (Nonius, 2000 ▶); cell refinement: *DENZO*/*SCALEPACK* (Otwinowski & Minor, 1997 ▶); data reduction: *DENZO*/*SCALEPACK*; program(s) used to solve structure: *SIR2004* (Burla *et al.*, 2005 ▶); program(s) used to refine structure: *SHELXL97* (Sheldrick, 2008 ▶); molecular graphics: *DIAMOND* (Brandenburg, 2008 ▶); software used to prepare material for publication: *SHELXL97*.

## Supplementary Material

Crystal structure: contains datablock(s) I, global. DOI: 10.1107/S1600536811033794/jh2317sup1.cif

Structure factors: contains datablock(s) I. DOI: 10.1107/S1600536811033794/jh2317Isup2.hkl

Supplementary material file. DOI: 10.1107/S1600536811033794/jh2317Isup3.cml

Additional supplementary materials:  crystallographic information; 3D view; checkCIF report

## Figures and Tables

**Table 1:** Hydrogen-bond geometry (Å, °)

*D*—H⋯*A*	*D*—H	H⋯*A*	*D*⋯*A*	*D*—H⋯*A*
N1*B*—H1*B*⋯O1*A*^i^	0.954 (18)	1.802 (18)	2.7526 (16)	174.0 (15)
N1*A*—H1*A*⋯N2*B*^ii^	0.915 (19)	1.95 (2)	2.8544 (18)	171.5 (16)

Symmetry codes: (i) 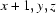; (ii) 
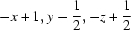
.

**Fig. 1.**
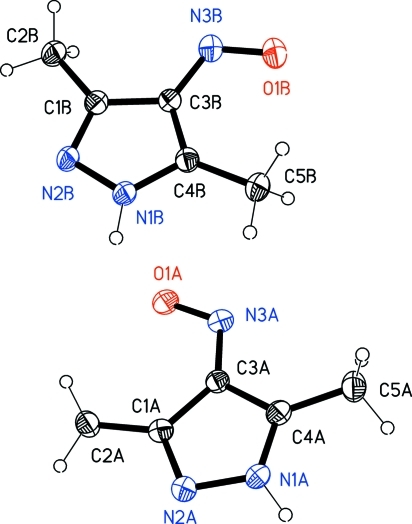
The two independent molecules of (I) in the unit cell, showing the atom numbering scheme.

**Fig. 2.**
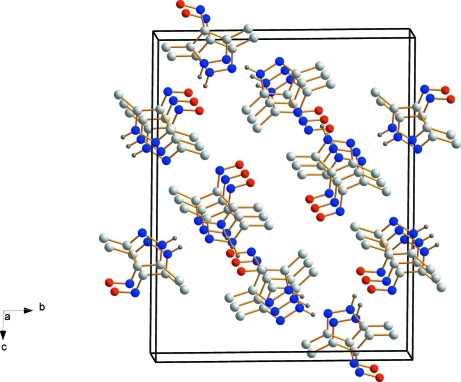
The crystal packing of the title compound showing the intermolecular hydrogen bonds by dashed lines.

## supplementary crystallographic information

### Comment

Pyrazole-based ligands are widely used in bioinorganic chemistry, molecular magnetism and supramolecular chemistry, as they are able to form different architectures, ranging from polynuclear clusters to metallocycles (Mullins, *et al.*, 2008; Mukhopadhyay, *et al.*, 2004). In addition to the ability to bridge two or more metal ions, pyrazole ligands also provide an effective magnetic exchange pathway between them (Aromi *et al.* 2006; Gatteschi, *et al.*, 2006). The incorporation of other coordinating groups to the pyrazole ring can increase the variety of polynuclear compounds that can be formed. For example, introduction of the potentially bridging oxime group in the molecules of the ligands already having bridging moieties (such as pyrazolates) can lead to increase of nuclearity and complexity of the metal complexes on their basis (Petrusenko *et al.*, 1997; Kanderal *et al.*, 2005; Sachse *et al.*, 2008; Moroz *et al.*, 2010). In this work, we report the crystal structure of the title compound which contains the oxime group in the 4-position of the pyrazole ring. Unlike 4-nitropyrazoles which have been widely used for preparation of oligonuclear metal complexes (Halcrow *et al.*, 2005), 4-nitrosopyrazoles have never been studied as ligands, and no metal complexes based on this type of ligands have been reported up to date. Crystal and molecular structures of only two 4-nitrosopyrazoles have been reported before (Cameron *et al.*, 1996; Fletcher *et al.*, 1997).

In the unit cell there are two types of conformers (A and B) of the title compound which differs significantly by the geometrical parameters and by the position of the oxime group with respect to the protonated pyrazole nitrogen (Fig. 1). In the conformer A, the oxime group is *trans*- with respect to the pyrazole hydrogen, while in the conformer B the oxime-group is *cis*-situated. In the conformers A and B the bond lengths markedly differs, first of all it is noticeable upon comparing the interatomic distances within the oxime groups. In the conformer B, the difference in bond lengths between C—N (1.3902 (19) Å) and N=O (1.2412 (16) Å) bonds of the oxime groups is quite large (*ca* 0.15 Å) while in the conformer A (C—N 1.3553 (19) Å and N=O 1.2701 (16) Å) it is much less pronounced (less than 0.08 Å). This clearly indicates that the CNO moiety in both conformers exists in the nitroso-form (Sliva *et al.* (1997); Mokhir *et al.*, 2002), however, in the conformer A there is a noticeable contribution of the isonitroso-form. Such a difference can be a consequence of the involvement of the oxime oxygen O1A in formation of the intermolecular H-bond, while O1B does not participate in any H-bond (Table 1).

The differences in geometrical and electronic structure of the oxime groups significantly influence on the C—C, C—N, N—N bond lengths within the pyrazole rings which are deviated from normal values (Kovbasyuk *et al.*, 2004; Wörl, Fritsky *et al.*, 2005; Wörl, Pritzkow *et al.*, 2005). Thus, there are signs of conjugation of the C(3B)—C(4B) bond with the O(1B)—N(3B) bond which results in noticeable shortening of the former (1.405 (2) Å) as compare to that observed in the conformer A, C(3)—C(4) = 1.442 (2) Å.

In the crystal, the molecules are linked by intermolecular N—H···O and N—H···N hydrogen bonds building zigzag chains along the *b* axis (Fig.2, Table 1). The translational along *a* axis chains form walls which are united into the crystal by van der Waals interactions.

### Experimental

3,5-dimethyl-4-nitrozo-1*H*-pyrazole was synthesized by using a literature procedure (Cameron *et al.*, 1996) from acetylacetone, sodium nitrite and hydrazine hydrate in aqueous hydrochloric acid. The crude product was collected by filtration and purified by recrystallization from benzene. Colorless crystals suitable for the X-ray diffraction were obtained after several hours (yield 78%).

### Refinement

The aromatic NH H atoms were located from the difference Fourier map and refined isotropically. Other H atoms were positioned geometrically and allowed to ride on their parent atoms, with C—H = 0.98 Å, and *U*_iso_ = 1.5 *U*_eq_ (parent atom).

### Crystal data

**Table:**

C_5_H_7_N_3_O	*F*(000) = 528
*M**_r_* = 125.14	*D*_x_ = 1.370 Mg m^−^^3^
Monoclinic, *P*2_1_/*c*	Mo *K*α radiation, λ = 0.71073 Å
Hall symbol: -P 2ybc	Cell parameters from 4238 reflections
*a* = 4.0268 (2) Å	θ = 1.0–27.5°
*b* = 15.3793 (7) Å	µ = 0.10 mm^−^^1^
*c* = 19.6627 (9) Å	*T* = 120 K
β = 94.613 (3)°	Plate, blue
*V* = 1213.75 (10) Å^3^	0.46 × 0.33 × 0.13 mm
*Z* = 8	

### Data collection

**Table:**

Nonius KappaCCD diffractometer	2747 independent reflections
Radiation source: fine-focus sealed tube	1866 reflections with *I* > 2σ(*I*)
horizontally mounted graphite crystal	*R*_int_ = 0.040
Detector resolution: 9 pixels mm^-1^	θ_max_ = 27.4°, θ_min_ = 2.5°
φ scans and ω scans with κ offset	*h* = −4→5
Absorption correction: multi-scan (*DENZO*/*SCALEPACK*; Otwinowski & Minor, 1997)	*k* = −18→19
*T*_min_ = 0.955, *T*_max_ = 0.987	*l* = −25→25
9003 measured reflections	

### Refinement

**Table:**

Refinement on *F*^2^	Primary atom site location: structure-invariant direct methods
Least-squares matrix: full	Secondary atom site location: difference Fourier map
*R*[*F*^2^ > 2σ(*F*^2^)] = 0.041	Hydrogen site location: inferred from neighbouring sites
*wR*(*F*^2^) = 0.109	H atoms treated by a mixture of independent and constrained refinement
*S* = 1.03	*w* = 1/[σ^2^(*F*_o_^2^) + (0.0516*P*)^2^ + 0.0988*P*] where *P* = (*F*_o_^2^ + 2*F*_c_^2^)/3
2747 reflections	(Δ/σ)_max_ < 0.001
175 parameters	Δρ_max_ = 0.23 e Å^−^^3^
0 restraints	Δρ_min_ = −0.25 e Å^−^^3^

### Special details

**Table:**

Geometry. All e.s.d.'s (except the e.s.d. in the dihedral angle between two l.s. planes) are estimated using the full covariance matrix. The cell e.s.d.'s are taken into account individually in the estimation of e.s.d.'s in distances, angles and torsion angles; correlations between e.s.d.'s in cell parameters are only used when they are defined by crystal symmetry. An approximate (isotropic) treatment of cell e.s.d.'s is used for estimating e.s.d.'s involving l.s. planes.
Refinement. Refinement of *F*^2^ against ALL reflections. The weighted *R*-factor *wR* and goodness of fit *S* are based on *F*^2^, conventional *R*-factors *R* are based on *F*, with *F* set to zero for negative *F*^2^. The threshold expression of *F*^2^ > σ(*F*^2^) is used only for calculating *R*-factors(gt) *etc*. and is not relevant to the choice of reflections for refinement. *R*-factors based on *F*^2^ are statistically about twice as large as those based on *F*, and *R*- factors based on ALL data will be even larger.

### Fractional atomic coordinates and isotropic or equivalent isotropic displacement parameters (Å^2^)

**Table:**

	*x*	*y*	*z*	*U*_iso_*/*U*_eq_	
O1A	−0.1796 (3)	0.16615 (7)	0.22546 (5)	0.0279 (3)	
N1A	0.3665 (3)	−0.05073 (9)	0.32527 (7)	0.0241 (3)	
N2A	0.4403 (3)	0.02548 (8)	0.36162 (6)	0.0243 (3)	
N3A	−0.1316 (3)	0.08489 (8)	0.22016 (7)	0.0240 (3)	
C1A	0.2710 (3)	0.08712 (10)	0.32773 (8)	0.0210 (4)	
C2A	0.2872 (4)	0.17879 (10)	0.35059 (8)	0.0258 (4)	
H2A	0.4408	0.1837	0.3917	0.039*	
H2B	0.0648	0.1981	0.3609	0.039*	
H2C	0.3667	0.2152	0.3144	0.039*	
C3A	0.0867 (4)	0.05016 (9)	0.26876 (8)	0.0202 (4)	
C4A	0.1598 (4)	−0.03950 (10)	0.27044 (8)	0.0225 (4)	
C5A	0.0466 (4)	−0.11093 (10)	0.22306 (9)	0.0308 (4)	
H5A	0.1902	−0.1133	0.1852	0.046*	
H5B	−0.1839	−0.1001	0.2052	0.046*	
H5C	0.0589	−0.1664	0.2476	0.046*	
O1B	−0.2132 (3)	0.13230 (7)	−0.04852 (6)	0.0356 (3)	
N1B	0.3699 (3)	0.21423 (8)	0.11942 (7)	0.0216 (3)	
N2B	0.3158 (3)	0.30207 (8)	0.10938 (7)	0.0226 (3)	
N3B	−0.1646 (3)	0.20960 (9)	−0.03253 (7)	0.0275 (3)	
C1B	0.1150 (4)	0.30759 (10)	0.05258 (8)	0.0214 (4)	
C2B	0.0044 (4)	0.39331 (10)	0.02337 (8)	0.0267 (4)	
H2B1	0.0884	0.4400	0.0540	0.040*	
H2B2	−0.2395	0.3953	0.0182	0.040*	
H2B3	0.0918	0.4009	−0.0213	0.040*	
C3B	0.0397 (4)	0.22318 (10)	0.02693 (7)	0.0201 (3)	
C4B	0.2123 (4)	0.16456 (10)	0.07173 (8)	0.0208 (4)	
C5B	0.2411 (4)	0.06867 (10)	0.07173 (8)	0.0264 (4)	
H5B1	0.3957	0.0502	0.1100	0.040*	
H5B2	0.3250	0.0494	0.0288	0.040*	
H5B3	0.0216	0.0428	0.0763	0.040*	
H1A	0.453 (4)	−0.1013 (13)	0.3437 (9)	0.040 (5)*	
H1B	0.515 (4)	0.1949 (11)	0.1572 (9)	0.035 (5)*	

### Atomic displacement parameters (Å^2^)

**Table:**

	*U*^11^	*U*^22^	*U*^33^	*U*^12^	*U*^13^	*U*^23^
O1A	0.0312 (6)	0.0231 (6)	0.0288 (7)	0.0049 (5)	−0.0017 (5)	0.0031 (5)
N1A	0.0283 (7)	0.0183 (7)	0.0252 (8)	0.0026 (6)	−0.0006 (6)	0.0023 (6)
N2A	0.0268 (7)	0.0221 (8)	0.0236 (7)	0.0000 (6)	0.0000 (6)	0.0000 (6)
N3A	0.0240 (7)	0.0244 (8)	0.0236 (7)	0.0010 (6)	0.0024 (6)	0.0034 (6)
C1A	0.0187 (8)	0.0230 (9)	0.0216 (8)	0.0002 (6)	0.0028 (6)	0.0019 (6)
C2A	0.0259 (8)	0.0246 (9)	0.0261 (9)	0.0004 (7)	−0.0024 (7)	−0.0031 (7)
C3A	0.0198 (8)	0.0204 (8)	0.0205 (8)	−0.0002 (6)	0.0023 (6)	0.0010 (6)
C4A	0.0224 (8)	0.0218 (9)	0.0235 (9)	−0.0005 (7)	0.0039 (7)	0.0021 (6)
C5A	0.0352 (9)	0.0228 (9)	0.0337 (10)	−0.0011 (7)	−0.0005 (8)	−0.0046 (7)
O1B	0.0459 (7)	0.0269 (7)	0.0328 (7)	−0.0057 (6)	−0.0044 (6)	−0.0041 (5)
N1B	0.0249 (7)	0.0172 (7)	0.0222 (7)	0.0014 (6)	−0.0014 (6)	0.0019 (5)
N2B	0.0270 (7)	0.0153 (7)	0.0250 (7)	0.0008 (5)	−0.0008 (6)	0.0011 (5)
N3B	0.0295 (7)	0.0247 (8)	0.0280 (8)	−0.0038 (6)	0.0007 (6)	−0.0014 (6)
C1B	0.0220 (8)	0.0203 (8)	0.0223 (8)	0.0005 (6)	0.0037 (7)	0.0001 (6)
C2B	0.0308 (9)	0.0205 (8)	0.0282 (9)	0.0013 (7)	−0.0010 (7)	0.0030 (7)
C3B	0.0212 (8)	0.0194 (8)	0.0198 (8)	−0.0005 (6)	0.0020 (6)	0.0002 (6)
C4B	0.0204 (8)	0.0211 (8)	0.0214 (8)	−0.0018 (6)	0.0041 (7)	−0.0013 (6)
C5B	0.0313 (9)	0.0184 (8)	0.0294 (9)	0.0012 (7)	0.0017 (7)	−0.0003 (7)

### Geometric parameters (Å, °)

**Table:**

O1A—N3A	1.2701 (16)	O1B—N3B	1.2412 (16)
N1A—C4A	1.319 (2)	N1B—C4B	1.330 (2)
N1A—N2A	1.3922 (18)	N1B—N2B	1.3801 (17)
N1A—H1A	0.915 (19)	N1B—H1B	0.954 (18)
N2A—C1A	1.3170 (19)	N2B—C1B	1.3279 (19)
N3A—C3A	1.3553 (19)	N3B—C3B	1.3902 (19)
C1A—C3A	1.442 (2)	C1B—C3B	1.417 (2)
C1A—C2A	1.479 (2)	C1B—C2B	1.492 (2)
C2A—H2A	0.9800	C2B—H2B1	0.9800
C2A—H2B	0.9800	C2B—H2B2	0.9800
C2A—H2C	0.9800	C2B—H2B3	0.9800
C3A—C4A	1.410 (2)	C3B—C4B	1.405 (2)
C4A—C5A	1.488 (2)	C4B—C5B	1.479 (2)
C5A—H5A	0.9800	C5B—H5B1	0.9800
C5A—H5B	0.9800	C5B—H5B2	0.9800
C5A—H5C	0.9800	C5B—H5B3	0.9800
			
C4A—N1A—N2A	113.82 (13)	C4B—N1B—N2B	113.61 (12)
C4A—N1A—H1A	129.1 (11)	C4B—N1B—H1B	126.7 (10)
N2A—N1A—H1A	116.9 (11)	N2B—N1B—H1B	119.7 (10)
C1A—N2A—N1A	105.42 (12)	C1B—N2B—N1B	105.14 (12)
O1A—N3A—C3A	115.11 (12)	O1B—N3B—C3B	115.32 (13)
N2A—C1A—C3A	109.56 (13)	N2B—C1B—C3B	109.83 (13)
N2A—C1A—C2A	121.60 (13)	N2B—C1B—C2B	121.54 (13)
C3A—C1A—C2A	128.84 (13)	C3B—C1B—C2B	128.62 (14)
C1A—C2A—H2A	109.5	C1B—C2B—H2B1	109.5
C1A—C2A—H2B	109.5	C1B—C2B—H2B2	109.5
H2A—C2A—H2B	109.5	H2B1—C2B—H2B2	109.5
C1A—C2A—H2C	109.5	C1B—C2B—H2B3	109.5
H2A—C2A—H2C	109.5	H2B1—C2B—H2B3	109.5
H2B—C2A—H2C	109.5	H2B2—C2B—H2B3	109.5
N3A—C3A—C4A	121.63 (13)	N3B—C3B—C4B	131.33 (14)
N3A—C3A—C1A	132.39 (14)	N3B—C3B—C1B	122.16 (14)
C4A—C3A—C1A	105.89 (13)	C4B—C3B—C1B	106.50 (13)
N1A—C4A—C3A	105.30 (13)	N1B—C4B—C3B	104.92 (13)
N1A—C4A—C5A	123.80 (14)	N1B—C4B—C5B	122.65 (14)
C3A—C4A—C5A	130.89 (14)	C3B—C4B—C5B	132.42 (14)
C4A—C5A—H5A	109.5	C4B—C5B—H5B1	109.5
C4A—C5A—H5B	109.5	C4B—C5B—H5B2	109.5
H5A—C5A—H5B	109.5	H5B1—C5B—H5B2	109.5
C4A—C5A—H5C	109.5	C4B—C5B—H5B3	109.5
H5A—C5A—H5C	109.5	H5B1—C5B—H5B3	109.5
H5B—C5A—H5C	109.5	H5B2—C5B—H5B3	109.5
			
C4A—N1A—N2A—C1A	0.03 (17)	C4B—N1B—N2B—C1B	−0.10 (17)
N1A—N2A—C1A—C3A	−0.15 (16)	N1B—N2B—C1B—C3B	0.46 (17)
N1A—N2A—C1A—C2A	−179.56 (13)	N1B—N2B—C1B—C2B	−178.40 (13)
O1A—N3A—C3A—C4A	178.61 (13)	O1B—N3B—C3B—C4B	2.4 (2)
O1A—N3A—C3A—C1A	2.4 (2)	O1B—N3B—C3B—C1B	−179.01 (14)
N2A—C1A—C3A—N3A	176.84 (15)	N2B—C1B—C3B—N3B	−179.59 (13)
C2A—C1A—C3A—N3A	−3.8 (3)	C2B—C1B—C3B—N3B	−0.8 (2)
N2A—C1A—C3A—C4A	0.22 (16)	N2B—C1B—C3B—C4B	−0.65 (17)
C2A—C1A—C3A—C4A	179.57 (15)	C2B—C1B—C3B—C4B	178.11 (15)
N2A—N1A—C4A—C3A	0.11 (17)	N2B—N1B—C4B—C3B	−0.30 (16)
N2A—N1A—C4A—C5A	179.38 (14)	N2B—N1B—C4B—C5B	178.69 (13)
N3A—C3A—C4A—N1A	−177.26 (14)	N3B—C3B—C4B—N1B	179.36 (15)
C1A—C3A—C4A—N1A	−0.19 (16)	C1B—C3B—C4B—N1B	0.56 (16)
N3A—C3A—C4A—C5A	3.5 (3)	N3B—C3B—C4B—C5B	0.5 (3)
C1A—C3A—C4A—C5A	−179.39 (16)	C1B—C3B—C4B—C5B	−178.29 (15)

### Hydrogen-bond geometry (Å, °)

**Table:**

*D*—H···*A*	*D*—H	H···*A*	*D*···*A*	*D*—H···*A*
N1B—H1B···O1A^i^	0.954 (18)	1.802 (18)	2.7526 (16)	174.0 (15)
N1A—H1A···N2B^ii^	0.915 (19)	1.95 (2)	2.8544 (18)	171.5 (16)

Symmetry codes: (i) *x*+1, *y*, *z*; (ii) −*x*+1, *y*−1/2, −*z*+1/2.
